# Therapeutic Uses and Efficacy of Low-Dose Naltrexone: A Scoping Review

**DOI:** 10.7759/cureus.81086

**Published:** 2025-03-24

**Authors:** Kayla K Leiber, Robert W Parker

**Affiliations:** 1 Medicine, Alabama College of Osteopathic Medicine, Dothan, USA; 2 Pharmacology, Alabama College of Osteopathic Medicine, Dothan, USA

**Keywords:** chronic pain management, fibromyalgia and inflammation, hailey-hailey disease, ldn, low-dose naltrexone, opioid alternative, opioid-free analgesia

## Abstract

Low-dose naltrexone (LDN) has been suggested as a novel treatment option for several conditions and is of increasing interest due to its potential ability to address certain medical conditions that lack effective treatments or frequently rely on the use of opioids as treatment. This article will synthesize evidence and assess the scope of literature that examines low-dose naltrexone’s efficacy against painful and other relevant medical conditions. A scoping review was conducted using the Preferred Reporting Items for Systematic Reviews and Meta-Analyses Extension for Scoping Reviews (PRISMA-ScR) guidelines. Resources utilized for the review included PubMed and Excerpta Medica database (Embase). Articles included were required to be original research, published in English, conducted on human subjects, published in the last 15 years, have full text available, and include treatment with naltrexone in an off-label capacity (i.e., not for the treatment of alcohol or opioid use disorder). Zotero (Corporation for Digital Scholarship, Vienna, VA), a reference management software, was used to review all search results with this criterion. Search results yielded 2,399 articles; 995 did not meet inclusion criteria, 1,166 were excluded after screening by title, and 142 were excluded after screening by abstract. Ultimately, 68 articles were included after a full-text review. The articles selected presented clinical examples of LDN efficacy for a variety of medical conditions. These articles also helped to illuminate the current gaps in research, pointing to the need for larger clinical trials and proper dosing studies.

## Introduction and background

Alternative treatments to opioids are of increasing importance and relevance for managing chronic pain [1]. Despite over 50 million Americans experiencing chronic pain each year, there are still minimal efficacious, inexpensive, and non-addictive treatment options [2]. Naltrexone, a mu-opioid receptor antagonist, is an FDA-approved medication primarily used at a 50 mg dose for the treatment of opioid and alcohol use disorder. In recent years, interest has grown in low-dose naltrexone (LDN), typically administered at 1 mg to 6 mg, for its potential anti-inflammatory and analgesic effects beyond its established role in addiction treatment. Research suggests that LDN may act as a glial cell modulator by antagonizing toll-like receptor 4, leading to anti-inflammatory and immunomodulatory effects [3]. Naltrexone at low doses, known as LDN, is becoming increasingly used and studied for its efficacy against various pain-related or painful conditions, certain cancers, and immune dysregulation disorders. Low-dose naltrexone is also inexpensive and is generally well tolerated with minimal side effects [3].

The efficacy of LDN has been most commonly reported in preclinical and clinical studies examining the treatment of fibromyalgia, multiple sclerosis, complex regional pain syndrome (CRPS), various gastrointestinal conditions (including Crohn’s disease), dermatological conditions (such as Hailey-Hailey disease), and certain cancers [4]. However, studies continue to emerge that analyze the effect of LDN against a variety of conditions, even extending beyond chronic painful and autoimmune conditions. The clinical use of LDN remains off-label, with no standardized, regulatory-approved treatment protocols.

This scoping review sought to establish a basis of the current knowledge of LDN based on current studies available and identify the gaps in current research. This review focused on the studies completed in the last 15 years and specifically focused on which medical conditions have been studied against LDN and what the efficacy was amongst these various conditions.

## Review

Methods

The protocol used for this review followed the Preferred Reporting Items for Systematic Reviews and Meta-Analyses Extension for Scoping Reviews (PRISMA-ScR) Checklist [5]. Inclusion criteria required the article to be original research, published in English, conducted on human subjects, published in the last 15 years, had full text available, and included treatment with LDN in an off-label capacity (i.e. not for the treatment of alcohol or opioid use disorder). PubMed and Excerpta Medica database (Embase) were used to select the articles. The search terms and automation tools are summarized in Table 1.

**Table 1:** Publication search strategy Embase: Excerpta Medica database

Search Engine	PubMed
Search terms	“Low dose naltrexone” OR “Low-dose naltrexone”
Publication date	2009-2024
Language	English
Search Engine	Embase
Search terms	'low dose naltrexone' OR (low AND ('dose'/exp OR dose) AND ('naltrexone'/exp OR naltrexone))
Publication date	2009-2024
Language	English

**Figure 1:**
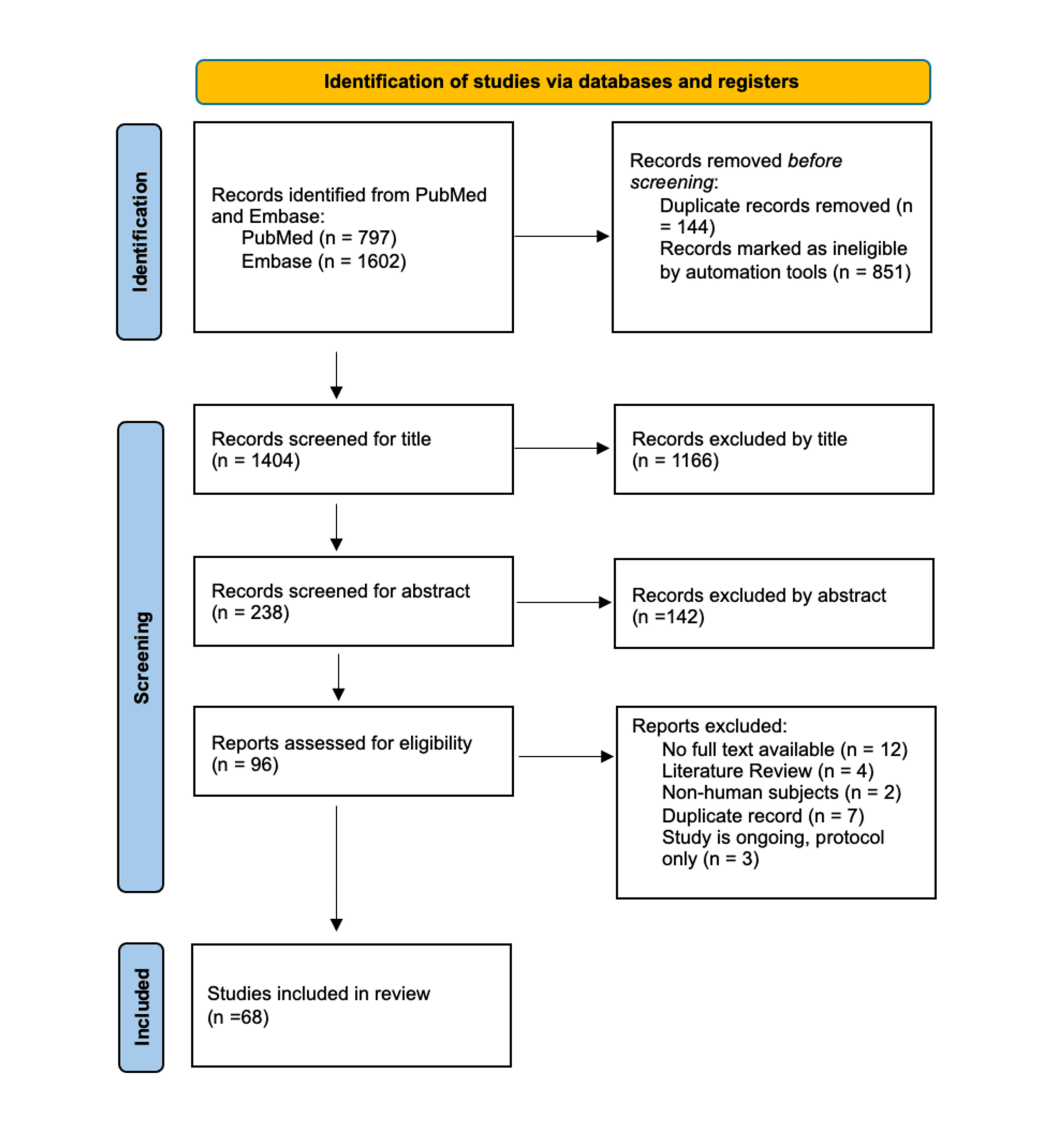
PRISMA flow diagram PRISMA: Preferred Reporting Items for Systematic Reviews and Meta-Analyses; Embase: Excerpta Medica database

The initial search resulted in 797 PubMed results and 1,602 Embase results. Zotero (Corporation for Digital Scholarship, Vienna, VA) was used to manage all article results, and any duplicates between the databases were removed from the initial search. Following the removal of duplicates with Zotero as well as the use of automation tools for initial screening, 1,404 articles were included for further screening. These articles were then screened by title, eliminating articles that did not align with the subject matter of this scope, which yielded 238 results. The articles were then screened by abstract, further eliminating studies that did not observe LDN efficacy or treatment, which yielded 96 articles. These 96 articles were given a full-text review and assessed for eligibility. Following a full-text review, 68 articles were included in the final selection. The selection process resulting in the final 68 articles is summarized in Figure 1. Additionally, the 68 final articles are summarized in Table 2, which emphasizes the purpose of the research study, the LDN protocol used, and the conclusion gathered from the study.

**Table 2:** Articles meeting all criteria and included after a full-text review Embase: Excerpta Medica database

Author	Title	Purpose	LDN Protocol	Conclusion	Database
Albers et al., 2017 [6]	Treatment of Hailey-Hailey Disease With Low-Dose Naltrexone (LDN)	To determine whether LDN is an effective treatment for Hailey-Hailey disease	Low-dose naltrexone, 3 mg nightly, titrated to 4.5 mg nightly in 2 patients	Three cases are presented indicating successful treatment of severe Hailey-Hailey with LDN	PubMed
Beaudette-Zlatanova et al., 2023 [7]	Pilot Study of Low-dose Naltrexone for the Treatment of Chronic Pain Due to Arthritis: A Randomized, Double-blind, Placebo-controlled, Crossover Clinical Trial	To study the efficacy of LDN in reducing chronic pain in patients with osteoarthritis (OA) and inflammatory arthritis (IA)	4.5 mg LDN for eight weeks and placebo for eight weeks	Findings do not support LDN being efficacious in reducing nociceptive pain due to arthritis	PubMed
Beltran, 2019 [8]	Low-dose Naltrexone: An Alternative Treatment for Erythrodermic Psoriasis	To review the treatment of erythrodermic psoriasis with LDN	Three months of 4.5 mg of LDN daily	LDN has proven to be a great ally in treating erythrodermic psoriasis flare-ups as an alternative treatment with fewer side effects	PubMed
Bested et al., 2023 [9]	Low-dose naltrexone for treatment of pain in patients with fibromyalgia: a randomized, double-blind, placebo-controlled, crossover study	To examine if LDN is associated with analgesic efficacy compared with control in the treatment of patients with FM	Either LDN 4.5 mg or an inactive placebo was given orally once daily	Outcome data did not indicate any clinically relevant analgesic efficacy of the LDN treatment	PubMed
Bolton et al., 2020 [10]	Low-dose naltrexone as a treatment for chronic fatigue syndrome	To examine the response of treatment with LDN in patients who exhibit chronic fatigue syndrome	LDN ranged from 4 to 12 mg	Treatment showed a range of responses when taking LDN, from life-changing to a reduction in some symptoms	PubMed
Bonilla et al., 2023 [11]	Low-dose naltrexone use for the management of post-acute sequelae of COVID-19	To review LDN off-label use for the treatment of post-acute sequelae of SARS-CoV-2/long COVID	LDN dose ranged from 0.5 to 6 mg; the median dose was 2.0 mg	LDN was associated with improved clinical symptoms, fewer number of symptoms, and better functional status	PubMed
Bruun-Plesner et al., 2020 [12]	Low-Dose Naltrexone for the Treatment of Fibromyalgia: Investigation of Dose-Response Relationships	To explore dose-response relationships when treating fibromyalgia with low-dose naltrexone	LDN between 0.75-6 mg, and the dosing interval was 0.75 mg	11 of the 25 were classified as responders, reporting a minimum 30% decrease in pain from baseline	PubMed
Choksi et al., 2011 [13]	Low-Dose naltrexone (LDN) treatment for Crohn’s disease (CD): A tertiary care center experience	To report experiences utilizing LDN for patients with refractory Chron’s disease	4.5 mg LDN for 12 weeks	Two out of nine patients (22%) had subjective improvement in symptoms (less stools and abdominal pain)	Embase
Chopra et al., 2013 [14]	Treatment of complex regional pain syndrome (CRPS) using low-dose naltrexone (LDN)	To report the results of two cases with prominent CRPS symptoms treated with LDN	4.5 mg LDN per day (one dose at night)	There was remission of pain and dystonic spasms in Case 1, as well as remission of all CRPS symptoms (including fixed dystonia) in Case 2	PubMed
Codino et al., 2021 [15]	Low Dose Naltrexone in Conjunction with the Wahls Protocol to Reduce the Frequency of Chronic Migraines in a Patient With Multiple Sclerosis: A Case Study	To investigate the efficacy of LDN and the Wahls Protocol in reducing the frequency and severity of chronic migraine headaches in a 62-year-old White female with multiple sclerosis	LDN titrated to 4.5 mg nightly	LDN and dietary changes significantly improved the patient's quality of life by reducing the severity, duration, and frequency of her chronic migraine headaches	PubMed
Costa et al., 2023 [16]	Combination of Naltrexone and Isotretinoin for the Treatment of Darier Disease (DD)	To observe the effects of LDN on DD (given the similarity of DD pathogenesis with Hailey-Hailey disease)	LDN 4.5 mg/day while maintaining the isotretinoin therapy	After three months, there was a nearly complete clearance of the lesions, and no adverse effects were reported	PubMed
Cree et al., 2010 [17]	Pilot trial of low-dose naltrexone and quality of life in multiple sclerosis	To evaluate the efficacy of 4.5mg nightly naltrexone on the quality of life of multiple sclerosis patients	Eight weeks of LDN treatment with 4.5mg nightly naltrexone	LDN significantly improved mental health quality of life indices	PubMed
De Haar et al., 2012 [18]	Immunological changes in inflammatory bowel disease (IBD) patients responding to low-dose naltrexone treatment	To determine if LDN treatment affects various immune parameters in IBD patients	LDN 5 mg daily	Of the 20 IBD patients treated with LDN, eight (40%) showed a clinical response and all are in clinical remission	Embase
Dieckmann et al., 2021 [19]	Low-dose naltrexone is effective and well-tolerated for modulating symptoms in patients with neuropathic corneal pain (NCP)	To assess the efficacy and tolerability of LDN in refractory NCP patients	Oral LDN 4.5 mg at bedtime for at least four weeks	LDN was effective and well-tolerated for NCP treatment	PubMed
Driver et al., 2023 [20]	Efficacy of Low-Dose Naltrexone and Predictors of Treatment Success or Discontinuation in Fibromyalgia and Other Chronic Pain Conditions: A Fourteen-Year, Enterprise-Wide Retrospective Analysis	To investigate if patients have a perceived benefit of LDN in treating pain symptoms and to identify predictors associated with a perceived benefit or discontinuation of LDN	LDN ranged from 0.8 to 9.0 mg, while the most common dose was 4.5 mg once daily	65% reported benefit in their pain symptoms while taking LDN	PubMed
Due Bruun et al., 2024 [21]	Naltrexone 6 mg once daily versus placebo in women with fibromyalgia: a randomised, double-blind, placebo-controlled trial	To investigate whether 12-week treatment with 6 mg low-dose naltrexone was superior to placebo for reducing pain in women with fibromyalgia	LDN 6 mg for or placebo for 12 weeks	This study did not show that treatment with low-dose naltrexone was superior to placebo in relieving pain	PubMed
Frech et al., 2011 [22]	Low-dose naltrexone for pruritus in systemic sclerosis	To report changes in pruritus and total gastrointestinal tract (GIT) symptoms in patients taking LDN	LDN 2 mg for the first month, then 1 mg by mouth at bedtime each week up to 4.5 mg	There was an improvement in pruritus, and all patients had an improvement in total the University of California Los Angeles Scleroderma Clinical Trials Consortium Gastrointestinal Tract 2.0 (UCLA SCTC GIT 2.0) scores as well as in constipation and distention/bloating subscales	PubMed
Hamel et al,, 2023 [23]	Oral Low-Dose Naltrexone in the Treatment of Frontal Fibrosing Alopecia and Lichen Planopilaris: An Uncontrolled Open-Label Prospective Study	To investigate the use of low-dose naltrexone (3 mg oral daily) as adjunctive therapy in the treatment of fibrosing alopecia (FFA) and lichen planopilaris (LPP)	LDN at 3 mg for one year	The study supports further investigation of oral low-dose naltrexone as adjunctive therapy in the treatment of FFA and LPP if there is prominent erythema, and possibly scale	PubMed
Ibrahim et al., 2017 [24]	Low-Dose Naltrexone Treatment of Familial Benign Pemphigus (Hailey-Hailey Disease)	To assess low-dose naltrexone hydrochloride in the treatment of recalcitrant Hailey-Hailey disease	1.5 to 3.0 mg LDN per day	Each patient exhibited at least an 80% improvement, and all three patients had improvement in quality of life, with one reporting improvement in depression	PubMed
Idrisoglu et al,, 2016 [25]	The effect of low dose naltrexone on amyotrophic lateral sclerosis treatment	To investigate the efficacy and safety of naltrexone in the treatment of ALS	4-5 mg/day/kg LDN for six months	It was found that LDN was ineffective when combined with riluzole	Embase
Isman et al., 2024 [26]	Low-dose naltrexone and nicotinamide adenine dinucleotide (NAD+) for the treatment of patients with persistent fatigue symptoms after COVID-19	To assess whether LDN treatment with NAD + through iontophoresis patches could improve fatigue symptoms and quality of life after COVID-19	LDN 4.5 mg/day and supplementation with NAD + through iontophoresis patches	Data suggests treatment with LDN and NAD+ is safe and may be beneficial in a subset of patients with persistent fatigue after COVID-19	PubMed
Jahangiri et al., 2021 [27]	The efficacy of low-dose naltrexone as a therapeutic alternative for fibromyalgia (FM), chronic pain syndrome (CPS), and multiple sclerosis: A retrospective chart review	To identify if the median LDN therapeutic duration and dosage were different between FM, CPS, or multiple sclerosis patients, and to determine which disease group had an improvement in quality of life and pain alleviation	3.0-4.5 mg LDN/day	Data contributes to the ongoing clinical evidence of LDN efficacy with FM, multiple sclerosis, and CPS patients, 85.3% of FM and CPS patients improved or stayed the same	Embase
Kais, 2013 [28]	Low-dose naltrexone therapy improves active ulcerative colitis	To describe a case of active ulcerative colitis treated with LDN in a young adult	4.5 mg naltrexone/day	With LDN therapy, Crohn's Disease Activity Index (CDAI) scores decreased significantly, and improvement was recorded in both quality-of-life surveys	Embase
Khan et al., 2020 [29]	Efficacy of Low Dose Naltrexone in Psoriasis	To determine the efficacy of LDN in patients with psoriasis	One tablet of naltrexone 6mg daily	Low-dose naltrexone is an effective therapy for psoriasis as in other chronic dermatological diseases	PubMed
Lie et al., 2018 [30]	Low dose Naltrexone for induction of remission in inflammatory bowel disease patients	To investigate the potential of LDN to induce clinical response in therapy refractory IBD patients	4.5 mg naltrexone for 12 weeks	LDN-induced clinical improvement in 74.5%, and remission in 25.5% of patients	PubMed
Lim et al., 2020 [31]	Improvement in Hailey-Hailey disease with a combination of low-dose naltrexone and oral magnesium chloride: A case report	To report patient improvement in Hailey-Hailey disease following LDN and oral magnesium chloride	LDN 1.5mg/ day, titrated to 4.5 mg/day within six weeks and then at 6 mg/ day after six months combined with magnesium chloride	Treatment resulted in complete clearance of a Hailey-Hailey disease episode and quality of life was improved significantly	PubMed
Ludwig et al., 2016 [32]	Long-term treatment with low dose naltrexone maintains stable health in patients with multiple sclerosis	To report the effects of LDN treatment on patients diagnosed with multiple sclerosis	4.5 mg naltrexone nightly	Data suggest that LDN is non-toxic, inexpensive, safe, and if taken alone did not result in an exacerbation of disease symptoms	PubMed
Manuduhane et al., 2019 [33]	Low-dose naltrexone: a unique treatment for amyopathic dermatomyositis (ADM)	To present a patient with refractory ADM that was responsive to low-dose naltrexone therapy	LDN was gradually titrated to 4.5mg/day	Low-dose naltrexone adds a possible low-cost, limited-side-effect treatment option for our autoimmune dermatologic disease	PubMed
Marcus et al., 2024 [34]	Effective Doses of Low-Dose Naltrexone for Chronic Pain - An Observational Study	To perform an observational analysis to determine the range of effective naltrexone daily dosing in 41 patients with chronic musculoskeletal pain	Titration starting at 0.1 mg LDN per day with increases by 0.1 mg on every third day (6 mg maximum)	LDN with idiosyncratic dosing appears to effectively suppress chronic pain. Patients found success on a fixed titration schedule despite failed attempts in the past at a fixed 4.5 mg/day without titration	PubMed
McKenzie-Brown et al., 2023 [35]	Low-Dose Naltrexone (LDN) for Chronic Pain at a Single Institution: A Case Series	To review past experiences with patients prescribed LDN to see what types of painful conditions were most responsive to LDN in the patient population	Doses of LDN were titrated from 1 or 1.5mg up to 4 or 4.5mg daily	Patients with all types of neuropathic pain, including CRPS, were significantly more likely to have pain relief from LDN than patients with spondylosis (p=0.018)	PubMed
Melitas et al., 2015 [36]	An open-label trial of low-dose naltrexone (LDN) for Symptomatic Mesenteric Panniculitis	To evaluate naltrexone as an effective therapy for symptomatic mesenteric panniculitis	Oral low-dose naltrexone, 4.5 mg nightly, for 12 weeks	Low-dose naltrexone is safe and well tolerated in patients with symptomatic mesenteric panniculitis. In this study, two of three patients had symptomatic improvement with treatment	Embase
Metyas et al., 2018 [37]	Low Dose Naltrexone in the Treatment of Fibromyalgia	To investigate a possible adjunctive therapy for FM as a significant number of FM patients do not respond adequately to current drugs	LDN started at a dose of 3 mg at nighttime and could be titrated up to a maximum of 4.5 mg at nighttime	This study lends further support to the preliminary body of evidence that naltrexone is a well-tolerated and likely effective treatment option in the community setting	PubMed
Michael et al., 2020 [38]	Recalcitrant Hailey-Hailey Disease Successfully Treated with Low-dose Naltrexone	To present a case of recurrent painful and pruritic Hailey-Hailey disease treated with LDN	LDN 1.5mg at bedtime	The patient developed significant clinical improvement of the cutaneous lesions with LDN treatment after only 26 days of treatment	PubMed
Mischoulon et al., 2017 [39]	Randomized, proof-of-concept trial of low dose naltrexone for patients with breakthrough symptoms of major depressive disorder (MDD) on antidepressants	To examine LDN’s efficacy as augmentation for depressive breakthrough on pro-dopaminergic antidepressant regimens	Naltrexone 1mg b.i.d. or placebo	LDN augmentation showed some benefit for MDD relapse on dopaminergic agents. Confirmation in larger studies is needed	PubMed
Miskoff et al., 2018 [40]	Low Dose Naltrexone and Lung Cancer: A Case Report and Discussion	To present a case of a 50-year-old male with prolonged survival and a past medical history of prostate and lung cancer taking LDN	LDN 4.5 mg	In light of statistics and the patient’s comorbidities of chronic respiratory failure, asthma, and tracheostomy, his response to LDN is worth documenting and further investigating	PubMed
N Irwin et al., 2024 [41]	Efficacy and Safety of Low Dose Naltrexone for Chronic Pain	To evaluate the impact of LDN on pain and function in adults seen at a chronic pain clinic	LDN doses <10 mg for ≥1 month	LDN was associated with a statistically significant reduction in Pain, Enjoyment of Life, and General Activity (PEG) scores in adult chronic pain patients	PubMed
Nathoo et al., 2015 [42]	Low dose naltrexone in the treatment of Crohn's disease: A case series	To report a case series of IBD patients treated with LDN as an adjunct to conventional therapies	LDN 4.5 mg or 9 mg	IBD patients show clinical response to LDN (54%) and should be considered as an adjunct to conventional therapy or as a bridge therapy	Embase
O'Kelly et al., 2022 [43]	Safety and efficacy of low dose naltrexone in a long covid cohort; an interventional pre-post study	To explore the safety of LDN in patients with Post-COVID-19 Syndrome (PCS)	LDN 1 mg month one, 2 mg month two	LDN is safe in patients with PCS and may improve well-being and reduce symptomatology in this cohort	PubMed
Patel et al, 2019 [44]	Variable response of Hailey-Hailey disease to naltrexone: A case series	To add three cases of biopsy-proven Hailey-Hailey disease treated with naltrexone to the current literature	4.5 mg LDN nightly, increased to 12.5 mg in one patient	All patients showed variable responses to LDN. A 4.5-mg dose may not be sufficient, and 12.5-mg to 50-mg doses can be safely attempted for an adequate response	Embase
Paula et al., 2023 [45]	Association of low-dose naltrexone and transcranial direct current stimulation (tDCS) in fibromyalgia: a randomized, double-blinded, parallel clinical trial	To evaluate the analgesic and neuromodulatory effects of a combination of LDN and tDCS in patients with fibromyalgia	LDN 4.5 mg or placebo followed by five days of the drug combined with anodal tDCS	Combined LDN+tDCS has possible benefits in reducing pain frequency and intensity in fibromyalgia	PubMed
Peters et al., 2022 [46]	Effects of low-dose naltrexone on quality of life in high-grade glioma (HGG) patients: a placebo-controlled, double-blind randomized trial	To study whether LDN has an impact on the quality of life and fatigue in patients with high-grade glioma	LDN 4.5 mg orally at night or placebo	LDN has no effect on the quality of life and fatigue in HGG patients during concurrent chemotherapy and radiation therapy	PubMed
Ploesser et al., 2010 [47]	Low dose naltrexone: side effects and efficacy in gastrointestinal disorders	To determine the frequency of adverse effects of low-dose naltrexone in patients who have been treated for a variety of gastrointestinal disorders	Diarrhea: LDN 2.5 mg daily, constipation: 2.5 mg twice daily, and inflammatory bowel disease: 4.5 mg daily	Low-dose naltrexone appears to be helpful for a member of patients with gastrointestinal disorders	PubMed
Polo et al., 2019 [48]	Low-dose naltrexone in the treatment of myalgic encephalomyelitis/chronic fatigue syndrome (ME/CFS)	To report on the safety and effectiveness data accumulated in clinical practice when treating ME/CFS with low-dose naltrexone	LDN, 3.0–4.5 mg/day	This study showed a high frequency of treatment response to LDN and there was a good safety profile observed	Embase
Raknes et al., 2018 [49]	The Effect of Low-Dose Naltrexone on Medication in Inflammatory Bowel Disease: A Quasi-Experimental Before-and-After Prescription Database Study	To examine whether initiation of LDN by patients with IBD was followed by changes in dispensing of relevant medication	< 5mg LDN daily	The initiation of LDN was followed by reductions in the dispensing of IBD drugs with different mechanisms of action	PubMed
Raknes et al., 2017 [50]	Low dose naltrexone in multiple sclerosis: Effects on medication use. A quasi-experimental study	To study whether an increase in LDN use was followed by changes in dispensing of other medications used to treat MS	Median dose was 3.7 mg LDN	There was no difference in either dispensed cumulative doses or the number of prevalent users of MS-specific medication	PubMed
Raknes et al., 2019 [51]	Low dose naltrexone: Effects on medication in rheumatoid and seropositive arthritis. A nationwide register-based controlled quasi-experimental before-after study	To test the hypothesis that starting LDN leads to reduced dispensing of medicines used in the treatment of rheumatic disease	< 5mg LDN daily	Initiation of LDN therapy was followed by significant and clinically relevant reductions in cumulative dispensed dose or in the number of users of all examined medicines	PubMed
Rivera et al., 2019 [52]	Proceedings #47: Low Dose Naltrexone: A Viable Alternative for Long Term Chronic Pain?	To highlight a chronic pain patient who sustained a wean from high dose opioid regimen utilizing low-dose naltrexone	2.5mg naltrexone	LDN, as an adjunct to other non-opiate pain modalities, is a valuable addition to a multifaceted pain management approach	Embase
Ruhoy et al., 2022 [53]	Low-Dose Naltrexone and Pain Relief in Gadolinium Deposition Disease: A Case Series	To study GDD-related neuropathic pain response to LDN with previously inadequate response to standard pain regimens	LDN 1.5 mg at night, increased by 1.5 mg weekly to a maximum of 4.5 mg per day	These case results suggest that a well-designed, adequately powered, controlled trial of LDN in GDD patients is merited	Embase
Sangalli et al., 2023 [54]	Low-dose naltrexone for treatment of burning mouth syndrome (BMS)	To describe a new treatment modality (LDN) for BMS	LDN 3 mg	Results suggest that LDN may be a feasible and effective treatment for BMS, especially in patients refractory to traditional treatment	PubMed
Sharafaddinzadeh et al., 2010 [55]	The effect of low-dose naltrexone on quality of life of patients with multiple sclerosis: a randomized placebo-controlled trial	To assess the effect of LDN on the quality of life of patients with relapsing-remitting and secondary progressive multiple sclerosis	LDN 4.5 mg capsule for eight weeks followed by placebo for eight weeks	The study clearly illustrates that LDN is a relatively safe therapeutic option in relapsing-remitting and secondary progressive multiple sclerosis	PubMed
Siembida et al., 2022 [56]	Depression in Fibromyalgia Patients May Require Low-Dose Naltrexone to Respond: A Case Report	To illustrate our clinical experience that depressive disorders may require fibromyalgia to be treated with low-dose naltrexone before the depression goes into remission	Two months of naltrexone 4.5 mg twice a day	The case highlights the potential importance of using LDN in fibromyalgia patients with comorbid depression	PubMed
Smith et al., 2013 [57]	Safety and tolerability of low-dose naltrexone therapy in children with moderate to severe Crohn's disease: a pilot study	To evaluate the safety and tolerability of an opioid antagonist, naltrexone, in children with moderate to severe Crohn's disease	LDN (0.1 mg/kg) for eight weeks, followed by open-labeled treatment for eight additional weeks	Naltrexone therapy seems safe with limited toxicity when given to children with Crohn's disease and may reduce disease activity	PubMed
Smith-Pellegrin et al., 2021 [58]	Naltrexone, a therapeutic alternative in Darier disease	To present a patient with Darier disease successfully treated with naltrexone at low doses	LDN 1.5 mg every 24 hours for 15 days, then 2 mg every 24 hours for 15 days, then 4 mg daily for a month, and finally 12.5 mg every 24 hours for three months	Naltrexone showed a global improvement of 80% without side effects in a patient who had not presented improvement despite having received treatment for months with multiple therapies	Embase
Soin, 2021 [59]	Management of pediatric complex regional pain syndrome with low-dose naltrexone	To describe the use of LDN in an 11-year-old boy with CRPS	1 mg LDN for one month, titrated to 2.5 mg for three months, then 4 mg	The patient’s pain severity improved by 70%, and self-reported functional status improved by 60%	Embase
Sousa Gomes et al., 2020 [60]	Vulvar Hailey-Hailey disease treated with low-dose naltrexone: case report and literature review	To report a case of vulvar familial benign pemphigus, or Hailey-Hailey disease, treated successfully with low-dose naltrexone	LDN 3 mg nightly	Low-dose naltrexone may represent a cost-effective and successful treatment modality in nongeneralized Hailey-Hailey disease	PubMed
Srinivasan et al., 2021 [61]	Efficacy and safety of low-dose naltrexone in painful diabetic neuropathy: A randomized, double-blind, active-control, crossover clinical trial	To analyze the efficacy and safety of LDN on painful diabetic neuropathy	Either 2 mg naltrexone or 10 mg amitriptyline	Low-dose naltrexone exhibited similar efficacy and a superior safety profile compared with amitriptyline in painful diabetic neuropathy	PubMed
Stallkamp Tidd et al., 2023 [62]	Low-Dose Naltrexone Use in Postural Orthostatic Tachycardia Syndrome (POTS): A Case Series	To review the charts of six tilt table-confirmed patients with POTS who underwent a trial of LDN	Variable among each patient, between 1-4 mg LDN	Some patients noted benefit, but patient-reported outcome measures show a variable response profile	PubMed
Strazzulla et al., 2017 [63]	Novel Treatment Using Low-Dose Naltrexone for Lichen Planopilaris	To describe four LPP patients treated with LDN	LDN 3 milligrams per day	LDN provided benefits in all four patients including a reduction in symptoms of pruritus, clinical evidence of inflammation of the scalp, and disease progression	PubMed
Theriault et al., 2023 [64]	The Efficacy Of Low-Dose Naltrexone In Pediatric Chronic Pain: A Retrospective Analysis	To review the use of LDN in youth with chronic pain presenting to an outpatient pediatric pain clinic	LDN of unspecified dosage	LDN was demonstrated to improve pain and pain burden in some youth with chronic pain with minimal side effects	Embase
Timoney et al., 2021 [65]	Prurigo excoriée treated with low dose naltrexone	To report a case of the effective use of LDN in the treatment of a case of acne excoriée and prurigo excoriée, previously refractory to a range of many treatments over 30 years of time.	3 mg LDN	LDN could potentially be a safe and effective treatment for acne excoriée or prurigo excoriée, both being very challenging conditions to treat	PubMed
Varghese et al., 2024 [66]	Low-Dose Naltrexone for Excoriation Disorder	To provide a case presentation of a patient with excoriation disorder who benefited from LDN	Naltrexone 4.5 mg per oral (PO) once a day (QD)	The patient experienced a therapeutic benefit from naltrexone for her skin-picking disorder, as demonstrated by the temporal changes in her symptoms	PubMed
Vrooman et al., 2017 [67]	Therapeutic value of naltrexone as a glial modulator	To present clinical data from a heterogenic group of 110 patients treated with LDN	LDN doses between 1 and 4.5 mg/day	Substantial benefits in terms of pain reduction, improved cognitive function, and, occasionally, primary condition regression were noted with LDN	Embase
Weinstock, 2022 [68]	Efficacy of low dose naltrexone in patients with Crohn's colitis and Ileitis	To present two patients with symptomatic CD with a rapid clinical and endoscopic response to LDN	1 mg oral naltrexone daily which was increased to 4 or 4.5 mg daily	Both patients had a rapid clinical endoscopic response to LDN	Embase
Weinstock et al., 2020 [69]	Low-dose Naltrexone Therapy for Psoriasis	To present cases to further the evidence of the efficacy and safety of LDN in the treatment of psoriasis	4.5 mg of oral naltrexone	Marked improvement was seen in 53% of the 15 patients	PubMed
Younger et al., 2009 [70]	Fibromyalgia symptoms are reduced by low-dose naltrexone: a pilot study	To test the effectiveness of low-dose naltrexone in treating the symptoms of fibromyalgia	LDN 4.5 mg baseline (two weeks), placebo (two weeks), drug (eight weeks), and washout (two weeks)	Low-dose naltrexone reduced fibromyalgia symptoms in the entire cohort, with a greater than 30% reduction of symptoms over placebo	PubMed
Younger et al., 2013 [71]	Low-dose naltrexone for the treatment of fibromyalgia: findings of a small, randomized, double-blind, placebo-controlled, counterbalanced, crossover trial assessing daily pain levels	To test the impact of low-dose naltrexone on daily self-reported pain	LDN 4.5 mg/day	The preliminary evidence continues to show that low-dose naltrexone has a specific and clinically beneficial impact on fibromyalgia pain	PubMed
Zappaterra et al., 2020 [72]	Low-Dose Naltrexone reduces symptoms in Stiff-Person Syndrome (SPS)	To present a case of Stiff-Person Syndrome (SPS) that was responsive to LDN	LDN at an unspecified dosage	The study concludes that LDN may have some utility in treating and managing the symptoms of SPS	PubMed
Zashin, 2020 [73]	Sjogren's Syndrome and Clinical Benefits of Low-Dose Naltrexone Therapy: Additional Case Reports	To describe patients with Sjogren's Syndrome whose conditions responded favorably to treatment with LDN therapy	LDN between 1-3.5 mg	LDN may be a useful therapy to help with joint pain and other constitutional symptoms in patients with this disorder	PubMed

Results

The final articles selected, 68 in total, ranged greatly in terms of the medical condition(s) studied. To summarize, the included medical conditions and number of studies are listed in Table 3.

**Table 3:** Medical conditions and number of studies

Medical condition	Number of studies and reference numbers
Chronic pain (relating to various conditions)	Eight studies [20, 27, 34, 35, 41, 52, 64, 67]
Fibromyalgia	Eight studies [9, 12, 21, 37, 45, 56, 70, 71]
Hailey-Hailey disorder	Six studies [6, 24, 31, 38, 55, 60]
Multiple sclerosis	Five studies [15, 17, 32, 50, 55]
Crohn's disease	Four studies [13, 42, 57, 68]
Post-acute sequelae of COVID-19	Three studies [11, 26, 43]
Inflammatory bowel disease	Three studies [18, 30, 49]
Psoriasis	Three studies [8, 29, 69]
Chronic fatigue syndrome	Two studies [10, 48]
Arthritic conditions	Two studies [7, 51]
Chronic regional pain syndrome	Two studies [14, 59]
Darier disease	Two studies [16, 58]
Lichen planopilaris	Two studies [23, 63]
Amyotrophic lateral sclerosis	One study [25]
Ulcerative colitis	One study [28]
Mesenteric panniculitis	One study [36]
Gadolinium deposition disease	One study [53]
Neuropathic corneal pain	One study [19]
Systemic sclerosis	One study [22]
Amyopathic dermatomyositis	One study [33]
Major depressive disorder	One study [39]
Non-small cell lung cancer	One study [40]
High-grade gliomas	One study [46]
Various gastrointestinal disorders	One study [47]
Burning mouth syndrome	One study [54]
Painful diabetic neuropathy	One study [61]
Postural orthostatic tachycardia syndrome	One study [62]
Prurigo and acne excoriee	One study [65]
Excoriation disorder	One study [66]
Stiff-person syndrome	One study [72]
Sjogren’s syndrome	One study [73]

In the conclusions column of Table 2, 62 studies reported at least some degree of efficacy in at least one or more participants when using LDN as treatment. What was an indication of treatment effectiveness varied with the type of medical condition being observed, with some studies reporting treatment efficacy as a decrease in overall pain and other studies considering treatment efficacy as an improvement in symptoms specific to the studied medical condition. Treatment efficacy ranged from mild symptom or pain relief to complete symptom clearance or disease remission. For example, Bolton et al. reported that treatment with LDN showed a range of responses, from life-changing to a reduction in some symptoms [10]. The type of study also varied amongst the 68 studies. Thirty-two of the 68 studies were case reports or case series that focused on small populations of patients treated with LDN in a clinical setting. Due to their limited sample sizes, these studies provided restricted data, which influenced the scope of conclusions that could be drawn regarding LDN’s overall effectiveness but still contributed valuable insights to the broader body of research. With the exclusion of case reports and series, the remaining 36 studies included randomized trials, as well as prospective, retrospective, observational, and pilot studies. These 36 studies included larger populations and were able to provide more overall data regarding treatment with LDN. Vrooman et al. [67] looked at a large set of clinical data following LDN treatment for a variety of medical conditions. The results of this study were similar to those of numerous other studies; however, this study was able to extrapolate from a larger data set. This study reported substantial benefits in terms of pain reduction, improved cognitive function, and, occasionally, primary condition regression amongst several conditions following LDN treatment. Similarly, Driver et al. [20] observed LDN treatment against several conditions and reported that 65% of patients perceived benefit in terms of their pain symptoms in addition to other symptoms (e.g., fatigue, brain fog, sleep). Many of the articles reported similar conclusions following treatment with LDN. As apparent in the LDN protocol portion of Table 2, the majority of studies reported LDN usage between 1 to 6 mg, with 4.5 mg being the most commonly utilized dosage across all studies.

Of the 68 articles selected, six articles reported LDN as an ineffective treatment against the medical condition being studied. Beaudette-Zlatanova et al. studied both osteoarthritis and inflammatory arthritis, and the study concluded that there were too few patients enrolled to rule out modest benefits or to assess inflammatory or neuropathic pain [7]. Bested et al. looked at LDN efficacy against fibromyalgia and concluded that LDN had no clinically relevant analgesic efficacy [9]. Due Bruun et al. also studied LDN against fibromyalgia and concluded that LDN was not superior to placebo treatment but might improve memory impairment associated with fibromyalgia [21]. Idrisoglu et al. looked at LDN treatment for amyotrophic lateral sclerosis and found that while safe, LDN was ineffective when combined with riluzole [25]. Peter et al. studied high-grade glioma patients, reporting that LDN did not significantly impact QOL or fatigue but indicated a subset of patients with high levels of baseline fatigue had some benefit [46]. Raknes and Småbrekke investigated multiple sclerosis and aimed to determine whether prescribing LDN led to a reduction in the dispensing of other multiple sclerosis medications. They concluded that there was no change in non-LDN multiple sclerosis treatments prescribed [50]. With the exception of these six studies, the remaining 62 studies reported LDN to have some degree of efficacy against the studied disease state, reporting effects ranging from mild symptom relief to total disease remission. Of the studies reporting treatment efficacy, authors reported efficacy against fibromyalgia [12, 20, 27, 37, 45, 56, 70, 71], multiple sclerosis [15, 17, 27, 32, 55], and rheumatoid arthritis [51], contrasting with some of the previously mentioned studies that did not find LDN efficacious against these disease states. No other studies included in this review reported on amyotrophic lateral sclerosis, high-grade glioma, or osteoarthritis.

Discussion

While the majority of the studies in this review reported treatment efficacy with LDN, the results varied significantly across the studies. Some studies reported complete disease remission, while others only noted mild symptom relief in a small percentage of participants. One explanation for the variability in treatment efficacy across various studies may be due to LDN dosage and protocol. The most common dosage amount across all studies was 4.5 mg of naltrexone taken once per day; however, there was some variation in effective dosages, with the majority ranging between 1 mg to 6 mg. Some studies used titration, gradually increasing the dosage until reaching 4.5 mg or an alternate effective dosage amount [6, 15, 31, 34]. For example, Marcus et al. utilized titration, starting at 0.1 mg LDN per day with increases by 0.1 mg on every third day with a 6 mg maximum [34]. Contrastingly, several other studies started their patients on a 4.5 mg dosage without initial titration [13, 16, 26, 28]. Observing the studies reporting ineffective treatment with LDN, none reported the use of titration when describing their dosage protocol [7, 9, 21, 25, 46, 50]. Marcus et al. reported a significant variation in effective doses amongst its participants [34]. This study also reported that multiple patients were able to achieve an effective dose on a titration schedule, even after prior unsuccessful attempts with fixed or variable doses of LDN. Further studies may be needed to determine proper dosing strategies, which was echoed by several authors. Additionally, previous studies ruling out LDN as an effective treatment for certain disease states might warrant reevaluation if titration was not utilized.

While most studies kept the dosage of naltrexone between 1 mg to 6 mg, some studies utilized higher dosages, which were both well-tolerated and effective [10, 20, 42, 44, 58]. Patel et al. reported treatment success with the usage of naltrexone between 12.5 and 50 mg without adverse effects [44]. This study concluded that the most utilized dosage of 4.5 mg naltrexone previously put forth by other publications may not be sufficient. While this article focused on higher dosages used for the treatment of Hailey-Hailey disease, further studies may be needed to explore the potential usage of naltrexone at slightly higher dosages than what was studied in the past. The information presented by several articles indicates that proper dosing studies need to be performed to investigate the true therapeutic range of LDN [27, 34].

Most studies described LDN as well-tolerated with minor adverse effects. The most commonly seen adverse effects noted across studies appeared to be vivid dreams, insomnia, nausea, and headache [19, 42, 48, 64, 70]. These side effects typically could be described as minor or transient [70] and were described as relatively rare and usually tolerable amongst the reported cases. These side effects do not appear to be a deterrent or concern for future utilization of LDN.

While the data presented across 62 of the 68 studies indicated some degree of treatment efficacy with LDN, it is worth noting apparent trends amongst the studies and which studies provided novel information about LDN. Amongst these articles, many focused on dermatological conditions. This included Hailey-Hailey disorder, Darier disease, alopecia and lichen planopilaris, psoriasis, and prurigo/acne excoriée. In these studies, LDN resulted in clearance of the lesions, vesicles, or erythema [6, 23]. Another feature among some dermatological-based studies was decreased pruritis [23, 65]. Similarly, pruritic symptoms were treated effectively in two non-dermatological studies, one related to pruritis in amyopathic dermatomyositis [33], and one related to pruritis in systemic sclerosis [22]. Other major areas of focus included gastrointestinal disorders. This included studies looking at mesenteric panniculitis and inflammatory bowel disease (both Crohn’s and ulcerative colitis). Successful treatment with LDN presented as decreased stools and abdominal pain [13] or total disease remission [30].

Another area of focus that a few studies explored was psychiatric illness. This included major depressive disorder (MDD) and excoriation disease. The effect of LDN on MDD was some benefit for MDD relapse on dopaminergic agents [39]. In excoriation disorder, LDN resulted in decreased compulsion [66]. Other studies cited comorbid depression improving when using LDN to treat other conditions. Ibrahim et al. reported concurrent improvement in depression while treating Hailey-Hailey disorder with LDN [24]. Other studies using LDN for fibromyalgia found improvement in comorbid depression [45,56].

Two studies reviewed LDN efficacy against chronic fatigue syndrome, reporting a majority of study participants had a positive treatment response with improved alertness and cognitive function [10,48]. Post-acute sequelae COVID-19 shares similar symptoms with chronic fatigue syndrome. Newer studies and ongoing trials for LDN are focusing specifically on post-acute sequelae of COVID-19 due to its high prevalence, which is seen globally in 43% of individuals who have previously had acute COVID-19 [11]. 3 studies were included in this review that looked at post-acute sequelae of COVID-19. These studies reported decreased fatigue, normalized sleep patterns, and a better functional status when treating post-acute sequelae COVID-19 with LDN [11,26,43]. All three studies looking at post-acute sequelae of COVID-19 concluded that further studies with larger, randomized, and controlled trials are warranted to further explore LDN efficacy and could possibly investigate which subpopulations may benefit from treatment.

The remaining studies focused mostly on chronic pain disorders and autoimmune conditions. These articles highlighted successful clinical uses for these conditions. This included fibromyalgia, multiple sclerosis, chronic pain syndrome, chronic regional pain syndrome, stiff-person syndrome, Sjogren’s syndrome, neuropathic pain, burning mouth syndrome, and rheumatoid and seropositive arthritis. Several of these articles mentioned successful LDN treatment that resulted in the discontinuation of other medications; this even included opioids in some instances [49, 51, 52]. Isman et al. focused on fibromyalgia, multiple sclerosis, and chronic pain syndrome and encouraged future studies to continue to investigate LDN as an off-label, non-opioid analgesic for pain, anti-inflammation, and disease modification [27]. These data help contribute to the current knowledge of LDN usage for treating chronic pain disorders and autoimmune disorders. These studies also provide compelling evidence that might prompt further research and clinical usage of LDN and contribute to the mounting amounts of evidence for these conditions. Additionally, many clinical trials are ongoing and may provide some more insight into LDN upon publication. Notably, ongoing clinical trials, such as LDN for pain management in HIV patients (NCT05537935)[74], LDN for post-COVID fatigue (NCT05430152) [75], LDN for diabetic neuropathy (NCT04678895) [76], and LDN for vasculitis (NCT03482479) [77], are investigating LDN’s efficacy across various conditions, providing valuable data to further refine its therapeutic potential.

This scoping review is limited in that it was only reviewed on PubMed and Embase. This review may have also excluded relevant research when reviewing articles by title and abstract only. This review also may have excluded relevant data by limiting the database search to the last 15 years. Scoping reviews typically do not conduct a formal risk of bias assessment. However, it is still important to acknowledge potential limitations and biases within the included studies. Many studies, particularly case reports and small-scale trials, had limited sample sizes, restricting the generalizability of their findings. Additionally, some studies lacked control groups, increasing the risk of confounding variables influencing the results. The potential for publication bias is also present, as studies reporting positive outcomes may have been more likely to be published than those reporting null or negative results. Among the randomized trials, variations in study design, follow-up duration, and outcome measures further contributed to inconsistencies in reported efficacy. The lack of standardized dosing protocols across studies introduces another element of bias, potentially affecting the comparability of findings.

## Conclusions

These articles provide a compelling argument for future clinical usage and observation of LDN while also helping to establish areas where there is still room for further research and understanding. The wide range of medical conditions studied across all articles indicates both the multidisciplinary interest in and relevance of LDN. This also indicates that there may be room for further research on LDN efficacy against several medical conditions that have yet to be explored. The majority of articles presented real clinical evidence of successful LDN treatment against the studied medical conditions. While these articles provided compelling clinical examples, large, randomized, and controlled clinical trials are still lacking in the current literature on LDN. Ongoing clinical trials may provide more insight into LDN upon publication. Due to the absence of standardized treatment guidelines for LDN, these articles present a variety of dosage protocols. While further studies would be needed to determine proper dosing strategies, including dosage amount, frequencies, and duration, these articles provide examples of efficacious dosages of LDN that may aid in the assigning of protocols for future LDN use. Low-dose naltrexone was reported to be efficacious in many studies and generally well-tolerated, though some adverse effects were noted. Low-dose naltrexone could be a safe and cost-effective treatment option for a variety of medical conditions, even extending beyond chronic pain conditions or inflammatory conditions, as these articles highlighted.
